# Editorial: Digital participation and communication disorders across the lifespan

**DOI:** 10.3389/fpsyg.2024.1417994

**Published:** 2024-05-06

**Authors:** Hendrike Frieg, Petra Jaecks, Kristina Jonas

**Affiliations:** ^1^University of Applied Sciences and Arts Hildesheim/Holzminden/Göttingen, Faculty of Social Work and Health, Hildesheim, Germany; ^2^Bielefeld University, Faculty of Linguistics and Literary Studies, Bielefeld, Germany; ^3^Paderborn University, Institute for German Language and Comparative Literature, Paderborn, Germany

**Keywords:** participation, communication disorders, digital, digital participation, speech and language, ICF

According to the UN Convention on Rights of Persons with Disabilities (see Articles 29, 30), participation is a human right. In this sense, participation in family, work and cultural life, recreation, leisure, sport, and political and public life must be the primary goal when empowering people with speech and communication impairments. As a consequence, improving all types of participation is the central goal of speech and language therapy. One major challenge of increasing and rapid digitization is ensuring digital participation for people with a variety of life situations and preconditions. While the original definitions of digital participation come from pedagogy and educational science, we see the need to further develop these specifications from the perspective of those affected by speech, language, and communication disorders. To date, the literature typically describes three main aspects of digital participation (Bosse, 2016; Bosse and Sponholz, 2023): (a) participation IN digital technologies, having access to and the ability to competently use digital devices, (b) participation THROUGH digital technologies, which entails participation through alternative access options, and (c) participation WITHIN the digital world, which means actively contributing to social networks, digital services, and media. In our view, this differentiation of digital participation seems inappropriate for the heterogeneous field of speech, language, and communication disorders and the people affected. Impairments in speech, language, and communication may occur across the lifespan at any age due to various etiologies. The commonality among such impairments is their impact on a person's ability to function with regard to speech, language, and communication, thereby affecting their activities and social participation. Thus, from our point of view, it seems worthwhile to use WHO's International Classification of Functioning, Disability and Health (ICF, World Health Organization, 2001) as a basis to clarify the different components involved.

The ICF serves as an international standard for framing, describing, and measuring functioning and disability. The individual characterization of the ICF components (body structures and functions, activities, and participation) covers all impacts of an existing health condition and should be accompanied by considerations of environmental and personal factors.

With respect to speech, language, and communication disorders, mental functions serve as the underlying basis, in the ICF coding especially those classed under b167 (*mental functions of language: reception, expression, and integrative language functions*). Furthermore, communication depends on *voice and speech functions* (b310–b340). These functions can be disturbed by many different health conditions, for e.g., congenital disabilities (such as autism spectrum disorder), developmental disorders, and neurological impairments (such as traumatic brain injury, stroke, or degenerative diseases). Furthermore, impairment to body structures in the brain (s110), mouth, tongue, throat, or ear (s310-s340; s240-260) might result in speech, language, and communication disorders.

Activities in the ICF framework refer to tasks or actions individuals perform that describe their general ability or competence in performing a specific task (in contrast to performing that specific task in everyday life). In the context of speech, language, and communication disorders, this means that activities linked to any form of communication are burdened.

Participation, considered the most important ICF component, analyzes how individuals live their life and incorporate their abilities into performing activities in daily living. The distinction between activities and participation is crucial because even if individuals are able to perform a task, they might feel too burdened or disabled by environmental or societal factors to perform the activity in their life as they wish to, although it is meaningful to them. Environmental or personal factors can then be examined separately to describe facilitators and barriers to performing activities in daily life, which impact individuals' participation. Considering communication disorders (ICF coding d3), disturbances are experienced when “communicating by [oral] language, signs and symbols, including receiving and producing messages, carrying on conversations, and using communication devices and techniques” (World Health Organization, 2001, p. 133). Products or technology for communication (e125) can facilitate communication, but access to alternative communication software and computer proficiency are considered critical factors.

As we move toward digitization, the scope of activities linked to (analog) communication is expanding. For example, when communicating with colleagues becomes difficult due to neurological speech impairment (dysarthria) which makes it impossible to use the telephone effectively, messaging services will represent a digital option that enables this activity, as reading and writing are unimpaired. Another example is the digital read-aloud function of many internet browsers, which allows individuals with a reading disorder (dyslexia) to engage in the activity of understanding online texts without reading.

At this point, it is important to stress that digitization has brought new forms of communication and correspondingly new activities to perform, for example, the activity of *forming relationships* (ICF code: d7200), which has always been dependent on language functions and communicative activities. Thanks to digitization, people experience new ways of forming relationships: they exchange information through internet forums or social media, maintain their relationships via messaging services or get to know each other using dating apps. *Forming relationships* has thus become a digital activity and must be considered a part of (digital) participation when dealing with individuals with speech, language, and communication disorders. Depending on the exact form of impairment, this expansion of participation can represent an opportunity (compensating for previous limitations and enabling participation) or a challenge (adding to the burden of severe limitations in participation).

Until recently, it did not seem necessary to differentiate between digital and analog aspects of participation, as the analog world was usually more important to many people. However, the digital world is increasingly gaining significance that has resulted in a separate type of participation known as digital participation.

Nevertheless, digital participation often remains a side issue. For example, digital participation is not coded in the ICF, although there are so many digitized areas of life. Therefore, when we look at the ICF from a therapeutic perspective, we should consider not only which activities beyond communication could be impaired due to the linguistic-communicative limitations of our clients but also whether these activities belong to the digital or analog world, or both (see Figure 1).

**Figure 1:**
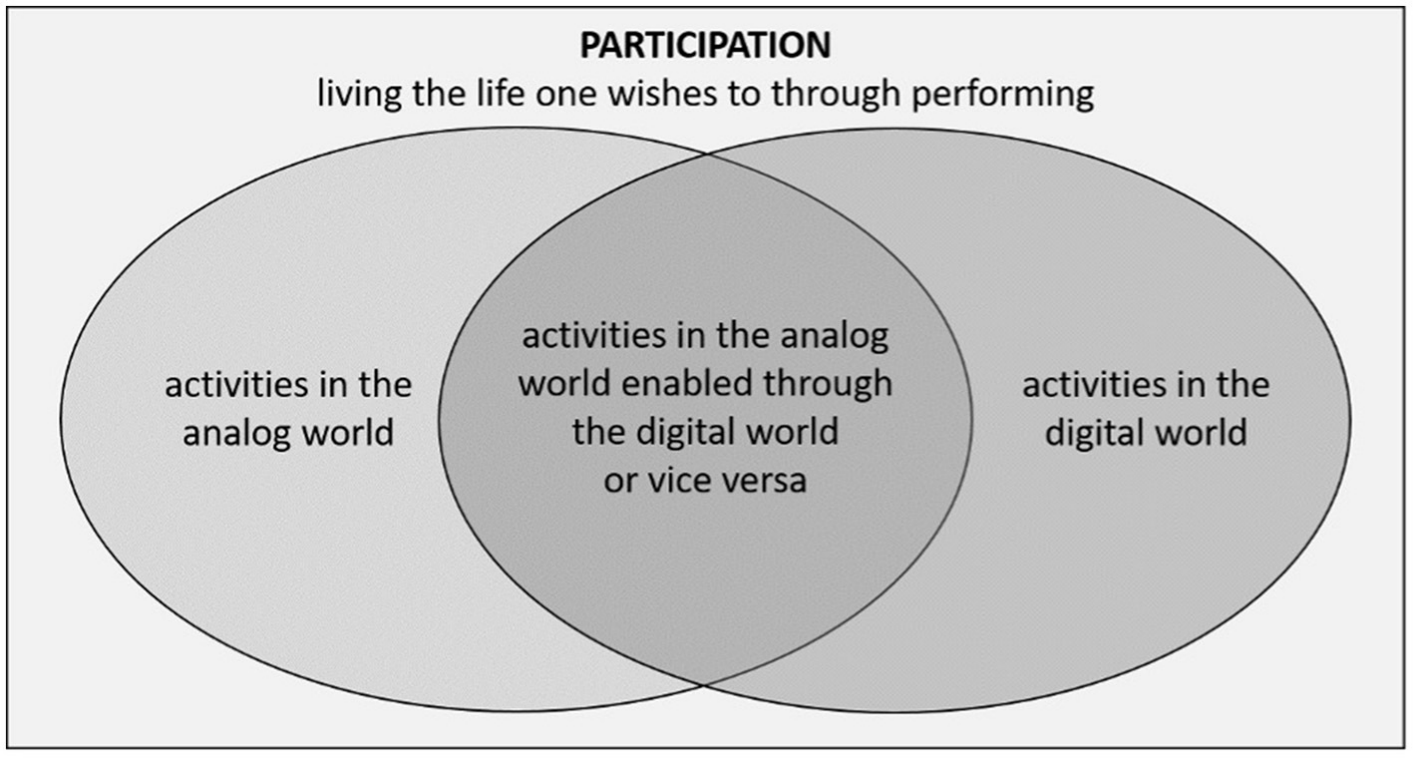
Digital activities in participation.

As shown in Figure 1, the activities overlap: using dating apps might lead to forming social connections, which in turn may result in engagement and participation in the analog world. However, this usage may also lead to digital interactions, leading to activities and participation in the digital world.

In our view, merely having access to technical devices, the internet, or a therapy app does not constitute digital participation for people with communication impairments. We prefer to speak of digital participation when people can perform activities relevant to them in the digital world, with all its social and technical possibilities, in the way and to the extent they wish to.

In this Research Topic, we see a wide range of activities that are relevant to digital participation. The ICF categorizes activities into nine major chapters: learning and applying knowledge (d1), general tasks and demands (d2), communication (d3), mobility (d4), self-care (d5), domestic life (d6), interpersonal interactions and relationships (d7), major life areas (d8), and community, social and civic life (d9). Analog and digital activities in all categories are important to people with speech, language, and communication disorders. Accordingly, activities from these categories can also be found in this Research Topic's articles.

Barthel et al. focus on activities in the d1 category by looking at decision-making in video-based telepractice as part of a qualitative analysis (d177 making decisions).

Both Wahl and Weiland, in their review of augmentative and alternative communication, and Keeley and Bernasconi, in their analysis paper, look at basic activities such as purposeful sensory experiences (d110-d129) as well as communication in particular, for example, producing non-verbal messages (d335).

Núñez Macías et al. analyzed the use and acceptance of voice assistants among people with aphasia, focusing on activities of communication through the use of telecommunication devices (d3600). Similarly, Azevedo et al. interviewed people with aphasia and their relatives on the use of communication aids (d360), relating to activities of communication (d3) and interpersonal relationships (d7).

The articles by Weiss et al., Büttner-Kunert et al., Ivarsson et al., Leinweber et al., and Heide et al. deal with activities of self-care, i.e.,. maintaining one's health (d5702). They focus on what digital speech, language, and communication therapy and diagnostics can look like and how they contribute to digital participation.

Kurfess et al. focused on peer-to-peer support through digital networking in individuals with aphasia, including activities such as engaging in social or community associations (d9100), while Pliska et al. and Schäfer and Miles present results on digital participation among individuals with autism spectrum disorder or those who are deaf or hard of hearing. Their studies also explore activities related to recreation and leisure (d920) as well as socializing activities (d9205).

Finally, Säuberli et al. involved people with intellectual disabilities in research, enabling them to actively exercise their right to autonomy and self-determination, thereby engaging them in activities concerning human rights (d940).

## Author contributions

HF: Writing – original draft, Writing – review & editing. PJ: Writing – original draft, Writing – review & editing. KJ: Writing – original draft, Writing – review & editing.
